# Review of "The Modern Technology of Radiation Oncology – A Compendium for Medical Physicists and Radiation Oncologists" by Jacob Van Dyk

**DOI:** 10.1186/1475-925X-5-5

**Published:** 2006-01-25

**Authors:** Michael Anbar

**Affiliations:** 1 Professor Emeritus, Amherst, NY, USA

This has been an unusually lengthy task reviewing these monumental two volumes of "The Modern Technology of Radiation Oncology" edited by Dr. Jacob Van Dyk (Medical Physics Publishing, Madison, WI), but it was also an enjoyable one. Although I have been teaching these topics for years, I was overwhelmed by the depth and breadth of most topics presented here, especially in the new second volume, some of which were new to me. To the best of my knowledge, these two volumes are the most comprehensive treatise on the subject written by an impressive group of international experts in the various domains of this broad field of clinical endeavor.

The first volume, published in 1999, also in a paperback edition, covers both theoretical and highly practical aspects of radiation oncology. The topics include detailed descriptions of practically all known treatment modalities: These range from skin treatments with electron beams, brachytherapy (use of implanted isotopic emitters), and internal radioisotope treatments (including the use of labeled antibodies), to kilovolt X rays, Co60 gammas, electron accelerators and megavolt treatments, to proton and neutron therapies. Separate chapters of this treatise also cover different treatment enhancements of radiation therapy, including photodynamic therapy, hyperthermia and boron neutron capture therapy. At the nuts and bolt level, separate chapters cover patient positioning and planning of treatment based on prior imaging (a more refined reiterative inverse planning modality is covered in a separate highly comprehensive chapter in Vol. II, published in 2005). The chapters in the second volume cover the use of Monte Carlo dose calculation in planning, intensity modulated therapy and different modeling techniques. These up to date chapters cover, in addition to general calibration techniques and the use of X-ray imaging for verification of positioning, also, specific topics such as breathing control during irradiation and brachytherapy of the prostate. In brief, I cannot think of a topic in radiation therapy of cancer that has not been covered comprehensively in one of these two volumes.

Like any other critical reviewer, I found in certain chapters a few minor mistakes, obscure passages, imprecise statements and certain minor oversights. However, when coming to write an all-encompassing review of this monumental compilation of information, I concluded that bringing up those details would be unwarranted nitpicking.

This is not a textbook for residents in this specialty when preparing for Board exams. Most topics are covered here in too much depth for that. Moreover, there is hardly a specialist in the field who is thoroughly versed in all the different modalities and techniques described here. The second volume is an up to date reference that will take more than a decade to become dated. I would expect, therefore that both volumes be part of the reference library of every radiation oncology department.

